# Pro-inflammatory CD8 T Cells Contribute to Age-Related Large Artery Stiffening

**DOI:** 10.1093/geroni/igaa057.440

**Published:** 2020-12-16

**Authors:** Daniel Trott, Sunita Sharma

**Affiliations:** 1 University of Texas at Arlington, Arlington, Texas, United States; 2 University of Texas at Arlington, ARLINGTON, Texas, United States

## Abstract

We have shown that T cells accumulate around the aorta with advanced age and that both pharmacologic and genetic deletion of total T cells result in improved large artery stiffness in old mice. The purpose of this study was to test the hypothesis that CD8 T cells specifically mediate age-related large artery stiffening. We randomized old (22-24 month) C57Bl6 mice (n=5/group) to treatment with an anti-CD8 or isotype control antibody (100µg every 5 days) for 28 days. We assessed large artery stiffness using pulse wave velocity (PWV) before and after antibody treatment. We found that PWV was similar between isotype (301±14 cm/s) and anti-CD8 (292±18 cm/s) before treatment. Following treatment, anti-CD8 treated mice exhibited lower PWV (272±11 cm/s) compared to the isotype (315±14 cm/s). Following euthanasia, we assessed aortic T cell infiltration by flow cytometry we found that anti-CD8 treated mice exhibited blunted aortic total CD3+ (262±42 vs. 1400±94 cells per aorta) and CD8+ (52±19 vs. 565±139 cells per aorta) cells compared to isotype controls. In a separate cohort of mice we compared interferon (IFN)-γ and tumor necrosis factor (TNF)-α production of aortic infiltrating CD8 cells from young (4-6 months, n=5) and old mice (n=8) using flow cytometry. A greater proportion of CD8 cells from old aortas produced IFN-γ (70±3% vs. 46±6%) compared to young. Similarly, a greater proportion of aortic infiltrating CD8 cells from old mice produced TNF-α (35±6% vs. 17±3%) compared to young. Collectively, these results suggest that pro-inflammatory CD8 cells contribute to cell non-autonomous arterial aging.

